# Gut Bacterial Diversity of Field and Laboratory-Reared *Aedes albopictus* Populations of Rio de Janeiro, Brazil

**DOI:** 10.3390/v15061309

**Published:** 2023-05-31

**Authors:** João M. C. Baltar, Márcio G. Pavan, Jessica Corrêa-Antônio, Dinair Couto-Lima, Rafael Maciel-de-Freitas, Mariana R. David

**Affiliations:** 1 Laboratório de Mosquitos Transmissores de Hematozoários, Oswaldo Cruz Institute, Oswaldo Cruz Foundation, Rio de Janeiro 21040-360, RJ, Brazil; joaomirandabaltar@gmail.com (J.M.C.B.); mgpavan@ioc.fiocruz.br (M.G.P.); jessicaacorrea@gmail.com (J.C.-A.); dcouto@ioc.fiocruz.br (D.C.-L.); freitas@ioc.fiocruz.br (R.M.-d.-F.); 2 Department of Arbovirology, Bernhard Nocht Institute of Tropical Medicine, 20359 Hamburg, Germany

**Keywords:** *Ae. albopictus*, gut microbiota, bacteria, Zika, 16S RNA gene sequencing

## Abstract

Background: The mosquito microbiota impacts different parameters in host biology, such as development, metabolism, immune response and vector competence to pathogens. As the environment is an important source of acquisition of host associate microbes, we described the microbiota and the vector competence to Zika virus (ZIKV) of *Aedes albopictus* from three areas with distinct landscapes. Methods: Adult females were collected during two different seasons, while eggs were used to rear F1 colonies. Midgut bacterial communities were described in field and F1 mosquitoes as well as in insects from a laboratory colony (>30 generations, LAB) using 16S rRNA gene sequencing. F1 mosquitoes were infected with ZIKV to determine virus infection rates (IRs) and dissemination rates (DRs). Collection season significantly affected the bacterial microbiota diversity and composition, e.g., diversity levels decreased from the wet to the dry season. Field-collected and LAB mosquitoes’ microbiota had similar diversity levels, which were higher compared to F1 mosquitoes. However, the gut microbiota composition of field mosquitoes was distinct from that of laboratory-reared mosquitoes (LAB and F1), regardless of the collection season and location. A possible negative correlation was detected between Acetobacteraceae and *Wolbachia*, with the former dominating the gut microbiota of F1 *Ae. albopictus*, while the latter was absent/undetectable. Furthermore, we detected significant differences in infection and dissemination rates (but not in the viral load) between the mosquito populations, but it does not seem to be related to gut microbiota composition, as it was similar between F1 mosquitoes regardless of their population. Conclusions: Our results indicate that the environment and the collection season play a significant role in shaping mosquitoes’ bacterial microbiota.

## 1. Introduction

*Aedes* mosquitoes are vectors of paramount relevance due to arbovirus transmission to humans [1,2]. *Aedes albopictus* has been implicated in dengue and chikungunya outbreaks in Europe, Africa and Asia [3,4,5,6]. It is also able to transmit at least 17 arboviruses under laboratory conditions, including Zika virus (ZIKV) and yellow fever virus (YFV) [7,8,9]. The successful establishment of this species is due to its ecological plasticity, which allows its survival in sylvatic and urban areas, where this mosquito blood-feeds on several vertebrate hosts and resists temperate climate; its larvae breed in natural and artificial water containers [7,8,10,11,12].

*Aedes albopictus* was first detected in Brazil in 1986, and since then, its local role as an arbovirus vector has been discussed [9]. Brazilian *Ae. albopictus* populations have low–moderate competence to ZIKV under laboratory conditions [13,14], and natural infections have been sporadically detected in field mosquitoes through the country [15,16]. Moreover, since it is a forest edge mosquito, it can potentially act as a bridge vector, carrying zoonotic pathogens from sylvatic animals to humans and vice versa [7,9,11]. This scenario raises the need to improve the knowledge about the ecology of *Ae. albopictus*, especially regarding vector competence to local arbovirus strains.

Among vector competence determinants, the host-associated microbiota has been pointed out as a modulator of arbovirus infection and transmission [17]. Some of the mechanisms by which the microbiota affects pathogen replication include the activation of vector immunity, competition with pathogens for resources and cell receptors in the midgut and the production of antiviral metabolites [18,19,20,21,22,23]. Thus, differences in microbiota composition can potentially explain the variations in vector competence usually observed among mosquito populations [24,25,26]. The microbiota can also influence mosquito development [27], blood digestion [28], nutrient acquisition [29] and peritrophic matrix synthesis [30].

The environment is largely indicated as a key factor shaping mosquito microbiota composition [31,32,33,34], as mosquitoes exhibit a similar microbiota when reared in the same breeding site or when collected in the same place [32,35,36]. Moreover, although some bacteria from the larvae microbiota can resist the metamorphosis to pupae and adult stages, the adult emerges with very few bacteria in the gut. Thus, its microbiota is mainly acquired from the environment by ingesting water from the breeding site [37] and exploring different food sources, such as floral nectar and blood meals on vertebrate hosts [38,39,40]. 

Therefore, we hypothesized that gut microbiota of natural *Ae. albopictus* populations vary according to the collection area, as well as to laboratory rearing/colonization. Thus, we investigated the gut bacterial diversity in field-collected and laboratory-reared (F1 and >F30 generations) adult females from three localities with different landscapes in Rio de Janeiro, Brazil, using Next-Generation Sequencing (NGS) of the 16S rRNA gene. Moreover, since gut microbiota can impact vector competence, we also orally infected mosquitoes with ZIKV and related body and head (i.e., dissemination) infection rates to potential variations in gut bacteria composition. 

## 2. Materials and Methods

### 2.1. Study Areas

**Figure 1:**
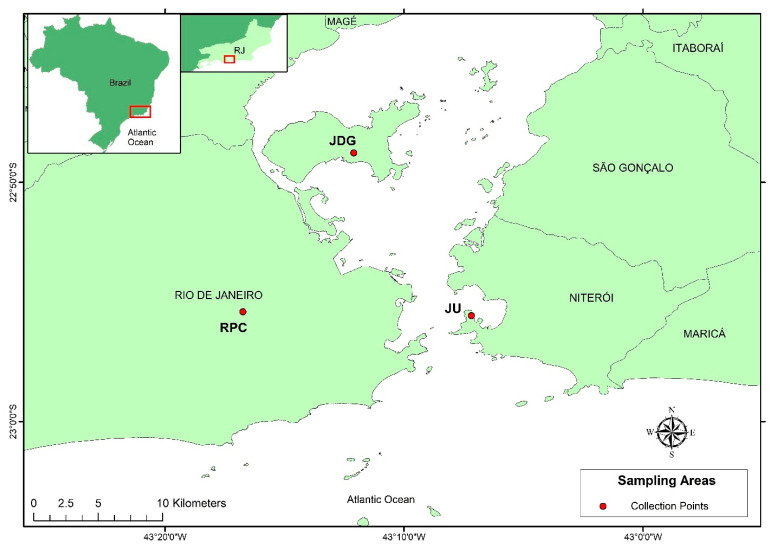
Mosquito collection areas. Map built using ArcGIS, sampling areas with different landscapes in Rio de Janeiro and Niterói cities. RPC—Represa dos Ciganos; JDG—Jardim Guanabara; JU—Jurujuba.

*Aedes albopictus* adult females and eggs were collected in three sites in Rio de Janeiro, Brazil [41,42]. Those localities were chosen due to their different landscapes: (1) Represa dos Ciganos (RPC), Rio de Janeiro, RJ (22°55′ S, 43°18′ O)—a forest area; (2) Jurujuba (JU), Niterói, RJ (22°55′ S, 43°06′ O)—a typical Brazilian slum—and (3) Jardim Guanabara (JDG), Rio de Janeiro, RJ (22°48′ S, 43°12′ O)—an urban neighborhood. The linear distance between these localities ranged from 16.5 to 20 km (Figure 1). Field collections were conducted in two different seasons in 2019: February (summer, wet season: 27.3 °C and 127.6 mm in Rio de Janeiro/109.8 mm in Niterói) and July to August (winter, dry season: 20.3 °C and 112 mm in Rio de Janeiro/71.6 mm in Niterói) [43].

### 2.2. Adult Collection and Identification

Adult mosquitoes were collected using backpack aspirators and transported alive to the laboratory in small cages. In JU and JDG, collections were conducted in the peridomestic area of 5–10 randomly selected premises in an area at least ~500 m long. All of them are close to vegetated areas, where it was possible to find *Ae. albopictus*. In RPC, collections were conducted along a trail in three fixed points: the beginning of the trail (forest edge), the middle (~500 m to 1 km from the edge) and at the end of the trail (~1.5–2 km from the edge). Adult mosquitoes were anesthetized on ice and taxonomically identified according to Consoli and Oliveira [44]. 

### 2.3. Egg Collection and Mosquito Rearing

Twenty ovitraps were installed in each collection point during the dry season of 2019. Their wooden paddles were replaced weekly until we obtained a minimum of 500 *Ae. albopictus* per sampled area. In the insectary (27 ± 2 °C and 70 ± 10% of relative humidity), paddles were submerged in 3 L of tap water in plastic trays containing 0.26 g of dry yeast. Larvae were fed daily with 0.45 g of Tetramin fish food (Tetra, Melle, Germany). Pupae were transferred to cages according to collection area and taxonomically identified using keys [44]. *Aedes albopictus* were fed with a solution of 10% glucose ad libitum and on anesthetized mice weekly for egg production (approved by the Fiocruz Ethical Committee for Animal Use—CEUA: L028/2018). Microbiota investigation and ZIKV infections proceeded with F1 mosquitoes (6–7 day-old) from each area reared and maintained following the same aforementioned protocol. 

### 2.4. Gut Processing and DNA Extraction

Microbiota diversity was individually investigated in field-caught adult *Ae. albopictus* collected during the wet and dry seasons of 2019 in each area or reared in the laboratory for one generation (F1). We also determined gut bacterial diversity of *Ae. albopictus* from a colony maintained for more than ten years in the laboratory (F > 30, LAB) to address the long-term effects of colonization on microbiota composition (Table 1).

Female mosquitoes were anesthetized on ice, surface disinfected with 70% ethanol for one minute and rinsed four times in sterile 1x phosphate-buffered saline solution (PBS). All dissection steps were performed using sterilized tweezers and a sterilized workbench. As a sterilization control for this process, we plated 100 µL from the last rinse in Luria–Bertani (LB) agar medium. The LB plates were incubated at room temperature for 48h to check for bacterial growth from the body surface of dissected mosquitoes, which did not occur. The guts were dissected under a Stereo Microscope (ZEISS), where mosquitoes were placed in a drop of 1x sterile PBS (approximately 8 µL) on a microscope slide. The midguts were removed and individually stored at −20 °C in 200 µL of PBS. Only insects with no apparent blood in the midgut were submitted to microbiota analyses.

Genomic DNA was extracted from individual midguts using the DNeasy Blood and Tissue Kit (Qiagen, Hilden, Germany) in a Biosafety cabinet (Trox^®^, Offenbach am Main, Germany) following the manufacturer’s instructions for Gram-positive bacteria. Blank samples were utilized as a negative control of the DNA extraction, which consisted of all reagents without *Ae. albopictus* midguts. In order to avoid any “batch effect”, samples had the DNA extracted in three different rounds of extraction, randomizing field, F1 and LAB gut samples [45].

### 2.5. Sequencing of the V3–V4 Region of the 16s rRNA Gene

Polymerase chain reactions (PCRs) amplified the V3-V4 hyper-variable region of the 16S rRNA gene using 341F (CCTACGGGNGGCWGCAG) and 805R (GACTACHVGGGTATCTAATC) primers [46] according to the 16S Metagenomic Sequencing Library Preparation protocol from Illumina [47]. Once individualized midguts had low microbial biomass [45], we increased the template DNA from 5 µL to 10.5 µL (1–3 ng of DNA), added 1 µL (10 mM) of forward and reverse primers (instead of 5 µL each) and performed 40 cycles of amplification. In addition to these modifications, we used 12.5 µL 2 × KAPA HiFi HotStart PCR mix (Roche, Switzerland) according to the manufacturer’s protocol [47]. Ultrapure distilled water (Gibco, Grand Island, NY, USA) with all PCR reagents and primers was used as negative control. In order to avoid any “batch effect”, samples had the 16S rDNA amplified in two different rounds, randomizing FIELD, F1 and LAB samples [45].

Samples (i.e., individual midguts) were purified using AMPure XP Beads and 16S libraries were prepared according to [47]. Library quantification was conducted using a Qubit^®^ 4 Fluorometer (Thermo Fisher Scientific, USA) and quality assessment was conducted using the Agilent TapeStation 4200. Paired-end (2 × 250 bp) sequencing was performed with an Illumina Miseq using kit MiSeq^®^v2 Reagent 500 cycles (Illumina Inc, San Diego, CA, USA). In total, 96 samples consisting of 94 individual midgut samples (detailed in Table S1) and 2 negative controls (1 pool of 3 extraction blanks and a pool of 2 PCR negative controls) were sent for 16s rRNA gene sequencing.

### 2.6. Bioinformatic Analysis

Illumina paired-end reads were demultiplexed in the Illumina BaseSpace Sequence Hub [48]. The QIIME2 (v. 2021.4) software [49] was used to process reads, infer the taxonomic affiliation and perform diversity and abundance analysis. The removal of sequence errors, index and adapters was performed with the cutadapt plugin [50], whereas quality control, chimera removal and dereplication were performed with the DADA2 plugin [51]. Taxonomic affiliation of amplicon sequence variants (ASVs) was inferred using the Greengenes 13_8 99% classifier [52,53]. Specifically for the reads assigned to the Acetobacteraceae family, we additionally used the BLASTN [54] and RDP classifier [55] for taxonomic identification. The dataset was submitted to a decontamination step using the microDecon package [56] in R (v. 4.1.1) to remove sequences considered to be cross-contamination (between biological samples) and contaminants from the laboratory environment, human manipulation [45], DNA extraction kit and PCR reagents (“kitome”) [57].

### 2.7. Diversity Analysis

Rarefaction curves were constructed to check the sampling depth and describe richness (number of ASVs) as functions of the sequences generated. Rarefaction was also used to equalize the number of sequences per sample, avoiding any bias due to variations in sequencing depth. The rarefaction threshold was established as 3800 sequences per sample. This value was chosen after the observation that the majority of samples reached a plateau in rarefaction curves, suggesting that the majority of bacterial taxa was detected (Figure S1). Four mosquito samples, one from groups RPC-F1 and JDG and two from JU-F1, with <3800 sequences were excluded from the dataset.

The analysis of microbial diversity was conducted using rarefied data through quantitative diversity indexes calculated from richness and abundance of ASVs (α diversity) and through a comparative matrix of microbiota composition between samples (β diversity) in QIIME2 [49]. Four α diversity metrics comprising estimates of richness, ecological diversity and equitability were calculated: observed ASVs, Shannon–Weaver index, Faith’s phylogenetic diversity and Pielou evenness. The results were visualized using boxplots generated using QIIME2 and the package qiime2R in R (v. 4.1.1) [58]. Differences in α diversity according to collection season, area, origin and population (detailed in Table 2) were investigated using the non-parametric Kruskal–Wallis test followed by pairwise comparisons, if necessary. The *p*-value was adjusted by the Benjamini–Hochberg multiple hypothesis false discovery rate (FDR) corrections for multiple comparisons (*q*-value) [59].

The β diversity was described through Bray–Curtis dissimilarity and Weighted Unifrac matrixes. Dissimilarities in microbiota composition between the aforementioned variables (Table 2) were visualized using Principal Co-ordinates Analysis (PCoA) plots generated using QIIME2 [49]. The β diversity indices were compared between groups using PERMANOVA (999 permutations) with a *p*-value < 0.05 considered statistically significant. In addition, we conducted pairwise PERMANOVA tests with *p*-values adjusted by the FDR (*q*-value) to detect significant differences in microbiota composition between the distinct mosquito groups. 

### 2.8. Taxonomic Composition, Differential Abundance Analysis and Core Microbiota

The relative abundance of bacteria was visualized through stacked barplots of the top 12 bacterial taxa at the lowest taxonomic level possible using qiime2R packages in R (v.4.1.1) [58]. Analysis of Composition of Microbiomes (ANCOM) [60] was employed to detect the differentially abundant bacterial taxa between mosquito groups (Table 2). As this method is sensitive to rare ASVs, ASVs with less than ten sequences and singletons (ASVs that occurred in only one sample from a group) were removed. Taxa were considered differentially abundant according to the W statistic, which indicates the number of times the null hypothesis was rejected for each taxon. The threshold for the W value is automatically determined by the qiime2 plugin according to dataset characteristics (e.g., the number of taxa and groups compared) [60].

The core microbiota was described as the ASVs (with at least ten reads) detected in at least 90% of host mosquitoes from a specific group [61]. The core was described considering all mosquitoes analyzed, sample origin and mosquito population (Table 2).

### 2.9. ZIKV Infection

*Aedes albopictus* oral infection was performed using F1 mosquitoes from the three populations with artificial feeders [62] using 2 mL of defibrinated rabbit blood, 1 mL of Leibovitz medium (L-15) and 1 mL of ~10^6^ PFU of a ZIKV strain isolated in Pernambuco, Brazil, during the 2015 outbreak of the infection (BRPE243/2015, Asian lineage) [63]. *Aedes aegypti* from the Urca neighborhood, Rio de Janeiro (AEG), was used as a positive control of virus infectivity for mosquitoes, since this vector population is known to be highly susceptible to ZIKV [64]. As a negative control, mosquitoes were fed with blood but with virus-free L-15 medium. Only visually blood-engorged females were selected for viral detection at 14 and 21 days post-infection (dpi) by RT-qPCR [65]. Viral copy number was determined in the mosquito’s body and head by absolute quantification using a standard curve with a seven-point serial dilution (10^1^–10^6^ copies) of an in vitro transcribed viral RNA [66]. Only samples with at least one thousand copies of ZIKV were considered positive. Infection rates (IRs) and dissemination rates (DRs) were calculated as the proportion of females with infected bodies among the total tested and the proportion of females with infected heads among those with infected bodies, respectively. The influence of *Ae. albopictus* population and dpi on IR and DR was estimated using logistic regression models and expressed as relative risks (RRs) and 95% confidence intervals (CIs). Differences in viral loads were compared using the Kruskal–Wallis test followed by the paired Dunn test, when necessary, applying the Bonferroni correction for multiple tests. The data from *Ae. aegypti* were not considered for the statistical analysis.

## 3. Results 

### 3.1. Sequencing Data

Illumina Miseq sequencing generated 4,169,369 raw reads, with an average of 43,431 reads per sample. Due to low Phred scores (<25) in most of the reverse sequences, we proceeded with the analysis with only the forward sequences. After the filtering and denoising steps, 1,871,448 sequences and 1619 ASVs were retained. The frequencies of filtered reads are described in Table S2. Decontamination retained 1,245,552 sequences, and seven ASVs were excluded as they were considered contaminants (Table S3). A dataset with 1612 ASVs was used for comparative analysis of gut bacterial diversity and composition.

### 3.2. Microbiota Diversity

#### 3.2.1. Alpha Diversity

**Figure 2:**
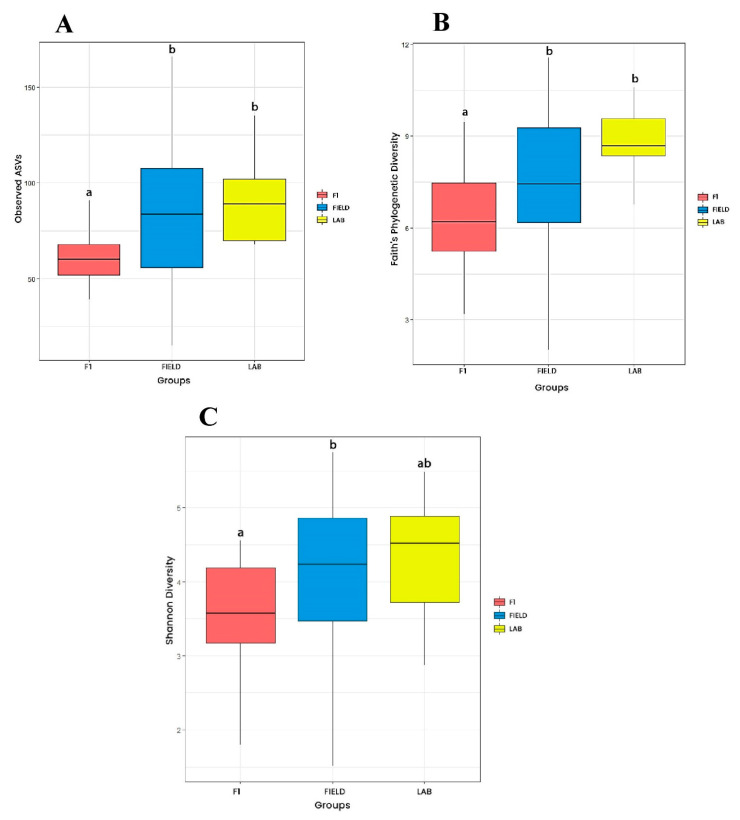
Alpha diversity of *Aedes albopictus* gut microbiota according to origin. (**A**) Observed ASVs (Kruskal–Wallis: H = 9.73; *p*-value < 0.001); (**B**) Faith (Kruskal–Wallis: H = 11.44; *p*-value < 0.05); (**C**) Shannon–Weaver (Kruskal–Wallis: H = 7.38; *p*-value < 0.05). Black lines indicate medians, and black dots indicate outliers. Groups indicated with the same letters are not significantly different (paired Kruskal–Wallis *q*-value > 0.05) (Table S4).

**Figure 3:**
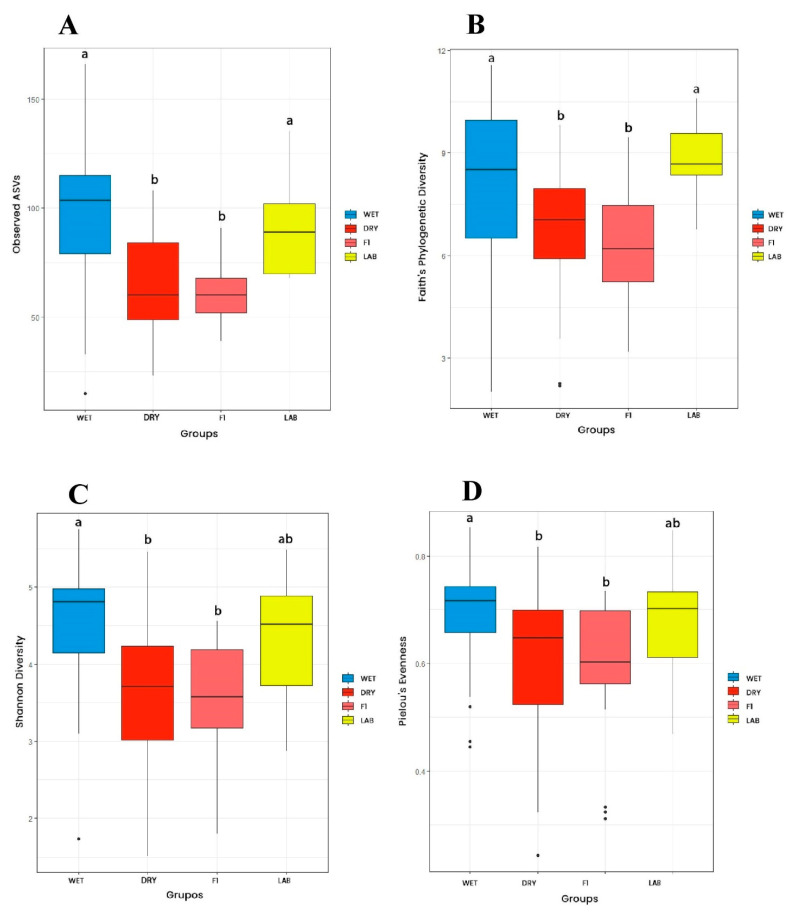
Alpha diversity of *Aedes albopictus* gut microbiota according to collection season. (**A**) Observed ASVs (Kruskal–Wallis: H = 29.60, *p*-value < 0.001); (**B**) Faith (Kruskal–Wallis: H = 17.58, *p*-value < 0.001); (**C**) Shannon–Weaver (Kruskal–Wallis: H = 21.94, *p*-value < 0.001); (**D**) Pielou (Kruskal–Wallis: H = 11.95, *p*-value < 0.05). Black lines indicate medians, and black dots indicate outliers. Groups indicated with the same letters are not significantly different (paired Kruskal–Wallis *q*-value > 0.05) (Table S4).

Regarding origin, Faith’s index and observed ASVs were higher for FIELD and LAB groups when compared to the F1 group (Figure 2A,B). The Shannon index was higher for FIELD in comparison to F1 samples, whereas LAB did not differ from both groups (Figure 2C). Considering collection season, all four indices were higher for the microbiota of *Ae. albopictus* from WET in comparison to those from the DRY season (Figure 3). Observed ASVs and Faith’s index were higher for LAB in relation to F1 mosquitoes (Figure 3A,B). In general, microbiota diversity did not vary significantly considering the collection area nor the population of *Ae. albopictus*. Significant differences were noticed only for the Faith’s index, with lower phylogenetic diversity of JDG-F1 when compared to LAB and RPC. Regarding population, a reduction in phylogenetic diversity was detected in JDG and JU mosquito microbiota in relation to the LAB group. Statistics can be found in figure legends, whereas pairwise comparisons for the four indices can be found in Table S4.

#### 3.2.2. Beta Diversity

**Figure 4:**
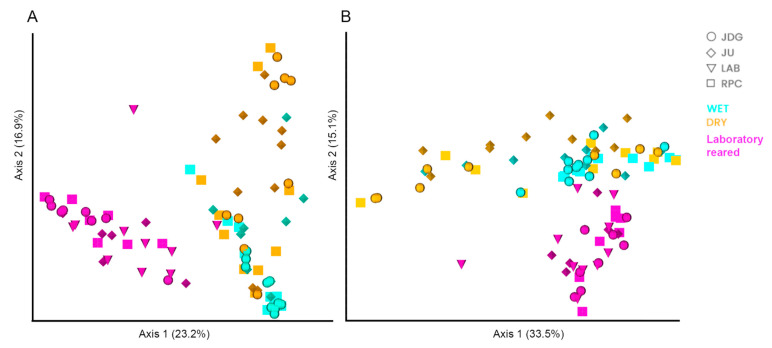
Principal component analysis (PCoA) of the gut microbiota of *Aedes albopictus*: (**A**) based on the Bray–Curtis dissimilarity matrix and (**B**) based on the Weighted Unifrac matrix. Shapes indicate the different populations, while colors show whether mosquitoes were collected during the wet or dry season or were laboratory reared (F1 and LAB). The variation explained by the PCoA axes is shown in parentheses.

The PERMANOVA analysis suggested a significant influence of all variables in gut microbiota composition, but pairwise comparisons only detected significant variations according to mosquito origin and collection season (Table S5), with a distinction between field vs. laboratory samples (F1 vs. FIELD Bray–Curtis PERMANOVA: pseudo-F = 20, *q*-value = 0.001; LAB vs. FIELD Bray–Curtis PERMANOVA: pseudo-F = 8.3, *q*-value = 0.001) and wet x dry season samples (WET vs. DRY Weighted Unifrac PERMANOVA: pseudo-F = 6.1, *q*-value = 0.001). The PCoA corroborates the pairwise test from PERMANOVA showing clustering between mosquito gut samples from the laboratory (F1 from the three field populations and LAB) versus those from field mosquitoes, regardless of collection area. Moreover, it is also possible to notice a cluster of samples from the WET season, whereas the DRY samples are more dispersed in the plot, suggesting a higher heterogeneity in the microbiota composition of mosquitoes from this group (Figure 4).

### 3.3. Aedes albopictus Microbiota Taxonomic Composition

**Figure 5:**
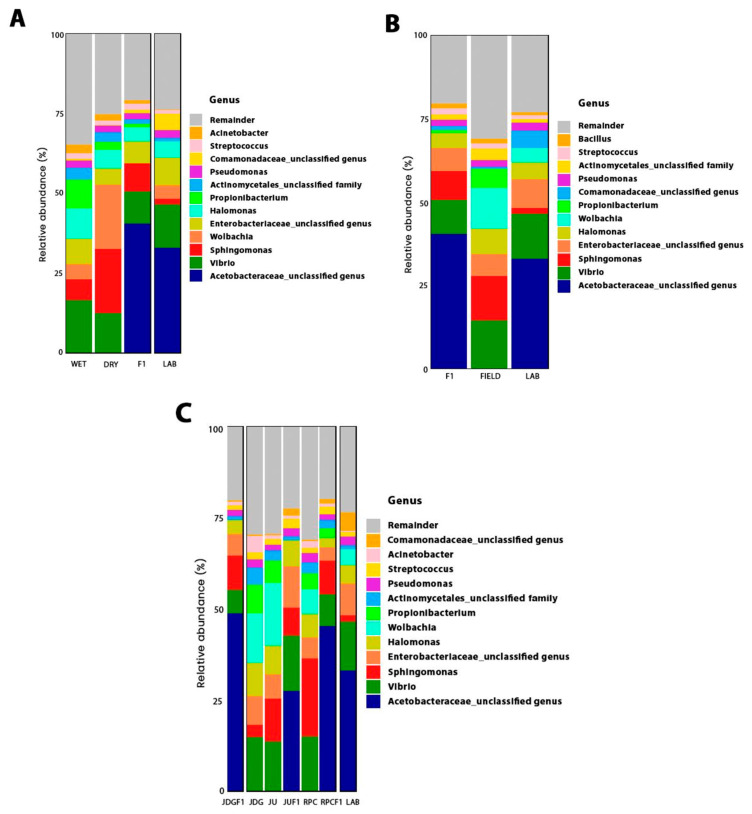
Relative abundance of ASVs, classified at the lowest taxonomic level possible according to (**A**) collection season, (**B**) origin and (**C**) collection area, detected in the midgut of mosquitoes from the groups described in Table 2. Legends at the upper right: the remainder belong to bacterial taxa with <3% of abundance (**C**).

In total, more than 100 bacterial genera were detected through 16S rDNA sequencing. However, the top 10 most abundant phylum and top 12 most abundant family/genera usually comprised >75% of sequences (Figure 5 and Figures S2 and S3). Proteobacteria was the predominant phylum in the microbiota of almost all *Ae. albopictus* females, with a mean relative abundance per group ranging from 66 to 88%, followed by Firmicutes, Actinobacteria and Bacteroidetes (Figure S2). Acetobacteraceae, *Vibrio*, *Wolbachia*, *Sphingomonas* and Enterobacteriaceae were, in general, the most abundant taxa in the midgut of *Ae. albopictus* (Figure 5). 

Acetobacteraceae was abundant only in laboratory mosquitoes (LAB and F1 of all populations), with an average abundance of 48, 45, 33 and 27% in JDG-F1, RPC-F1, LAB and JU-F1, respectively, and showed a much lower average abundance (<0.2%) in field-collected mosquitoes (JDG, JU and RPC) (Figure 5). In contrast, *Wolbachia* was detected in most field mosquitoes, regardless of the collection season, whereas it was only detected in three of ten samples from the LAB group (Figure 5). The presence of *Wolbachia* varied among field mosquitoes, but when detected, it was often one of the most abundant taxa. JDG had 6/19 samples with *Wolbachia,* with abundance ranging from 0.2% to 77.6% and an average abundance of 13.6% considering all gut samples. *Wolbachia* was detected in 14/19 of JU samples, varying from 0.8% to 55% of sequences (17.2% in average). The RPC group had the lowest *Wolbachia* average abundance (6.7%), with the widest variation in relative abundance between positive samples: 0.7% to 84.5%. This symbiont was detected in 7/20 of RPC samples. *Wolbachia* was more abundant in mosquitoes collected during the DRY season than those captured during the WET season (Figure 5). 

Based on PERMANOVA results, we ran ANCOM according to sample origin and collection season. The ANCOM for mosquito origin corroborated the presence of Acetobacteraceae, as well as *Rahnella* and an unclassified Alphaproteobacteria, as characteristic of F1 and LAB mosquitoes. Twelve of thirteen bacteria were considered differentially abundant in LAB samples, six of them exclusive of this group (Table 3). Regarding collection season, Acetobacteraceae was also differentially abundant taxa in F1, LAB and DRY samples (Table 4). *Wolbachia* was considered a signature of DRY, WET and LAB samples, whereas it was less abundant in the F1 group, reinforcing the results from Figure 5A (Table 4). Furthermore, *Ralstonia* and *Methylobacterium* were pointed out as typical microbiota from mosquitoes collected during the wet season (Table 4). 

### 3.4. Aedes albopictus Core Microbiota

*Aedes albopictus* core microbiota was composed of four bacterial families (Enterobacteriaceae, Moraxellaceae, Pseudomonadaceae and Halomonadaceae) and one order (Actinomycetales) (Table S6). The core microbiota of field mosquitoes (JDG, JU and RPC from both seasons) was composed of Bacillaceae, Propionibacteriaceae, Sphingomonadaceae, Streptococcaceae and Vibrionaceae, whereas the core microbiota of laboratory *Ae. albopictus* (F1 from all areas and LAB) was only composed of Acetobacteraceae. The JDG and JU core had Vibrionaceae as the most dominant bacterial taxa; the JU core had also Xanthomonadaceae, whereas the RPC core was composed only of Bacillaceae (Table S6).

### 3.5. Vector Competence to ZIKV

**Figure 6:**
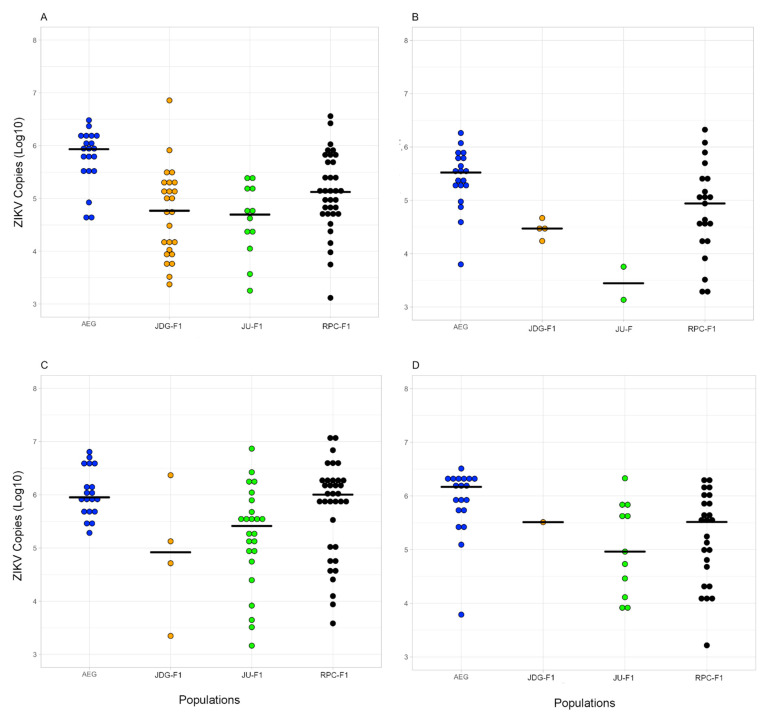
Quantification of ZIKV copies (log10) by RT-qPCR in the body (**A** for 14 dpi and **C** for 21 dpi) and head (**B** for 14 dpi and **D** for 21 dpi) of *Ae. albopictus*. The black horizontal line represents the median number of viral copies.

Approximately 600 *Ae. albopictus* females from each population and 200 from *Ae. aegypti* were exposed to ZIKV in two independent experimental infections. The infection rates ranged from 30 to 85.4%, while the dissemination rates ranged from 16 to 71.4% in *Ae. albopictus* (Table 5). Considering the IR, a significant population effect was observed on the proportion of infected insects (RR for JU: 0.42, RR 95% CI: 0.20–0.89, *p*-value = 0.02; RR for RPC: 3.97, RR 95% CI: 1.70–9.29, *p*-value = 0.001), whereas there was no difference between 14 and 21 dpi (RR: 1.02, RR 95% CI: 0.93–1.12, *p*-value = 0.61). Similarly, populations varied in DR, with markedly higher values for RPC at both dpi (RR for JU: 1.88, RR 95% CI: 0.53–6.63, *p*-value = 0.33; RR for RPC: 8.09, RR 95% CI: 2.65–24.74, *p*-value < 0.001). There was no significant difference between 14 and 21 dpi (RR: 1.09, RR 95% CI: 0.97–1.23, *p*-value = 0.13). In general, the number ZIKV copies was highly heterogeneous in the analyzed mosquitoes irrespective of their localities, with variations in up to three orders of magnitude. However, considering 14 and 21 dpi, there was no significant difference in viral load in the body and head of females between the three populations of *Ae. albopictus* tested (Kruskal–Wallis *p*-value > 0.05) (Figure 6A–D).

## 4. Discussion

*Aedes albopictus* gut microbiota diversity and composition was determined according to collection area and season, origin and population. Our results evidenced that bacterial diversity levels decreased when mosquitoes were reared for one generation in the laboratory (F1) in comparison to field-collected specimens, irrespective of *Ae. albopictus* population. Diversity was also lower in mosquitoes collected during the dry season in comparison to those from the wet season. On the other hand, diversity levels were similar between field *Ae. albopictus* and those from a lab colony with >30 generations in the insectary (LAB). Altogether, our data suggest that mosquito microbiota was mainly influenced by the environment, and the ZIKV infection and dissemination rates were not related to gut microbiota composition. 

Investigations on the diversity of the microbiota of *Ae. albopictus* have been executed worldwide, especially in Asia, Europe and the United States [67,68,69,70,71]. However, few were conducted in Latin America, including Brazil. At the phylum level, Proteobacteria, Firmicutes, Actinobacteria and Bacteroidetes were the most dominant phyla, as they often are for different mosquito species [27,35,36,72,73,74], including in adults and larvae of *Ae. albopictus* [36,75,76,77]. Proteobacteria was the most abundant phylum, regardless of whether the mosquitoes were from the field or laboratory, which is in line with what has been described for Chinese and Brazilian *Ae. albopictus* populations [68,76,78]. 

The variability in mosquito gut microbiota has been linked to larval breeding site and/or habitat factors [77]. Despite this, microbiota diversity and composition were not significantly different here between female *Ae. albopictus* collected in areas with different landscapes. Although geographical variations in *Ae. albopictus* microbiota composition have been described in Hawaii and China [68,79], other studies pointed to a weak or no contribution of collection area to diversity levels or microbiota composition of these insects [77,80,81]. For example, there was no correlation between microbiota diversity and the level of urbanization for populations from the island of O’ahu (Hawaii) [70]. On the other hand, the microbiota of *An. coluzzi* and *Ae. aegypti* were more diverse in mosquitoes collected in urban areas than in those collected in rural areas [82,83]. Such contrasting findings could be explained both by ecological and/or geographic factors. *Aedes albopictus* exhibits high ecological plasticity, colonizing a variety of natural and artificial breeding sites [7,8]. It is frequently found in urban environments and artificial containers in China [84], while it is considered a forest edge mosquito in Brazil [11]. Thus, the similarity between the microbiota of *Ae. albopictus* seen here could be due to similar breeding sites commonly available in vegetated areas from the three sampled sites. Moreover, the absence of a geographic signature in the microbiota could be also explained by the proximity of collection sites in this study (up to 20 km). In contrast, the gut microbiota of six different mosquito species, including *Ae. albopictus*, collected across eight areas separated from each other by at least 47 km in China, was correlated with geographic origin.

We observe a higher richness (observed ASVs), diversity (Faith, Shannon), evenness (Pielou) and heterogeneity in the microbiota of mosquitoes collected during the wet season than in those sampled during the dry season. This indicates a temporal instability of the mosquito microbiota, possibly by changes in climatic conditions and/or environmental microbes that can colonize the mosquito’s midgut [34,85]. Our experimental design cannot confirm these hypotheses, but laboratory experiments have indicated an increase in heat-tolerant taxa in the microbiota of mosquitoes submitted to higher environmental temperatures [86,87]. Monitoring the temperature and the microbiota of wild mosquitoes over time would help our understanding of the influence of this variable in mosquito-associated microbe diversity.

We noticed significant differences in the richness, diversity levels and composition of gut microbiota according to mosquito origin (field, F1 and LAB). This lower diversity and richness in the microbiota after laboratory colonization are in line with previous studies with *Ae. albopictus* [88,89,90]. The sudden change of environment from the field to the laboratory may have caused a remodeling in gut microbiota composition, as F1 and LAB specimens had a more similar gut microbiota composition to each other than to insects from the field (Figure 4). This microbiota shift might also have resulted from the loss of genetic variability after mosquito colonization [91] and/or standardized diet (10% glucose) and rearing conditions in the insectary. Even though wild mosquitoes were processed without visible blood in the midgut, we had no control over their diet in the field. Diet and blood sources affect gut microbiota diversity [92]: field-collected *Ae. aegypti* fed on human blood showed a greater diversity and richness in their microbiota compared to those that fed on non-human blood sources or were non-blood fed [93]. Moreover, we do not know the age of the wild mosquitoes, while the laboratory-reared specimens were processed at 6–7 days old. Age impacts the microbiota diversity of *Ae. albopictus*, favoring specific taxa according to the physiological state of the mosquito (newly emerged, young, older) [94].

Microbiota diversity levels were similar between wild *Ae. albopictus* and those from a colony with >30 generations of laboratory rearing (LAB) in terms of number of ASVs. Over generations, the insect gut tends to be colonized by bacterial taxa adapted to the type of food, rearing water and controlled temperature and humidity conditions of an insectary [94]. Thus, after some time, microbiota diversity would reach levels similar to field mosquitoes (but with a distinct composition). Studies comparing field populations with their respective laboratory colonies over several generations are needed to test this hypothesis and determine whether and when microbiota diversity levels would be restored. 

Changes in microbiota after mosquito colonization may have implications on vector competence studies. As microbiota can influence vector susceptibility to pathogens, the infection pattern of laboratory-reared mosquitoes might not reflect that of field mosquitoes. It is laborious to carry out vector competence experiments with wild-caught adult *Aedes* due to the large number of insects required [95], unknown mosquito age and physiological stage and low survival and blood-fed success caused by capture physical stress. An alternative way to produce mosquitoes with a more similar microbiota than their wild counterparts would be their rearing in the laboratory with natural breeding site water. This methodology allowed the conservation of ~50% of the bacterial families found in field mosquitoes in the microbiota of *An. gambiae* (F10) from Ghana [96].

There was a significant effect of *Ae. albopictus* population on the IR and DR of ZIKV, with markedly higher values for RPC at both 14 and 21 dpi. We have no evidence that this variation is related to the microbiota, since gut bacterial composition was similar between F1 mosquitoes from the three populations (Figure 4). However, we must consider that the microbiota was investigated in a sample of mosquitoes from the same batch of those exposed to experimental infections, i.e., we do not have ZIKV and microbiota information for the same specimens. Thus, we cannot exclude the possibility that the individual heterogeneity seen in gut bacterial composition may have impacted virus establishment in the vector. As we intended to identify possible gut symbionts related to virus establishment and dissemination in the mosquito, we opted to characterize the microbiota in mosquitoes before virus exposure, otherwise it would be elusive whether potential differences in gut bacterial diversity and composition between F1 mosquitoes from the three areas are a cause or consequence of ZIKV infection [97]. 

The interaction between microorganisms can modulate the microbiota composition of mosquitoes [88,98]. The Acetobacteraceae family of bacteria was more prevalent and relatively abundant in LAB and F1 mosquitoes, while *Wolbachia* was more prevalent and relatively abundant in FIELD mosquitoes, suggesting a possible co-exclusion interaction between these bacterial taxa. Acetobacteraceae are acetic acid bacteria (AAB), Gram-negative aerobic bacteria that carry out the oxidation of ethanol into acetic acid [99]. AAB are often found in insects with diets rich in sugar, colonizing different organs and tissues, including the midgut. They grow in acidic pH and produce an extracellular matrix of polysaccharides, which allow their contact with the intestinal epithelium without activating the host immune system [100]. Our laboratory colonies were fed with sugar solutions which were probably the source of acquisition and transmission of AAB between mosquitoes [101].

*Wolbachia* is an obligate intracellular bacterium that naturally colonizes >40% of insect species [102]. *Aedes albopictus* naturally harbors two *Wolbachia* strains, wAlbA and wAlbB [103], that are in higher density in the gonads than in the mosquito’s midgut [104]. A negative correlation between *Asaia* and *Wolbachia* was previously described in the reproductive tissues of *Ae. albopictus*, *Ae. aegypti*, *An. gambiae* and *Cx. quinquefasciatus* [105,106]. In our work, we used laboratory-reared females fed only with 10% glucose solution and processed at 6–7 days old. In this scenario, the molecular mechanisms by which these bacteria interact still need to be elucidated. 

Despite the possible co-exclusion interaction between Acetobacteraceae and *Wolbachia*, some LAB specimens exhibited a considerable abundance of both bacteria. This may be due to the longer period of colonization in the laboratory, which might have facilitated the establishment of new bacterial taxa from this environment in the gut and/or changes in the interactions between microorganisms, favoring the reestablishment of *Wolbachia* levels even in the presence of Acetobacteraceae. This hypothesis is supported by the higher diversity levels of LAB *Ae. albopictus* in relation to the F1, in which the increase in Acetobacteraceae possibly favored the decrease in *Wolbachia*. Nevertheless, the individual variation in *Wolbachia* relative abundance in mosquitoes from the same group could also be explained by host and/or environmental characteristics, as well as technical issues. We worked with the mosquitoes’ midguts, where *Wolbachia* density is usually low, which could explain why it was undetectable in some samples. In addition, the ages of field-collected mosquitoes could have influenced *Wolbachia* density, as older *Ae. albopictus* have lower densities of both wAlbA and B strains [107,108]. Although these *Wolbachia* strains are resistant to temperature increase [109] and there was no differential abundance of *Wolbachia* between the two collection seasons, environmental stresses from the field could also affect the endosymbiont density. Finally, even if we avoided blood-fed mosquitoes in which the microbiota multiplies up to a thousand times [110], fluctuations in gut microbiota load may have hindered the detection of *Wolbachia* in samples whose symbiont density was already low.

The core microbiota of adult *Ae. albopictus* from all groups was formed by the bacterial families Enterobacteriaceae, Halomonadaceae, Moraxellaceae and Pseudomonadaceae and the order Actinomycetales. Enterobacteriaceae comprises genera often associated with *Ae. Albopictus*, such as *Enterobacter*, *Klebsiella* and *Pantoea* [79,85,94,111]. Halomonadaceae was mainly from the *Halomonas* genus, a halotolerant bacterium (i.e., that survives in saline environments [112]) previously detected in the gut microbiota of field *Ae. albopictus* from the United States [88]. Pseudomonadaceae and Moraxellaceae families were mainly from *Pseudomonas* and *Acinetobacter* genera, respectively, which are also commonly found in the microbiota core of different mosquito vector species, such as *Ae. aegypti*, *Anopheles* spp. and *Ae. albopictus* [72,73,75,113,114,115]. Once acquired, *Pseudomonas* colonizes the intestine of mosquitoes and increases in abundance after blood-feeding [92,94], as it resists the oxidative stress caused by the heme from red blood cells [72]. It can be found in breeding water and in all stages of mosquito development, with evidence of transtadial transmission, since it was detected in the Malpighian tubules [116]. *Acinetobacter* is not only found in the gut of mosquitoes but also in breeding sites and food sources such as vertebrate host skin and plants. It may be involved with important functions for the physiology of *Ae. albopictus*, such as blood digestion and the assimilation of sugars from nectar [117]. Lastly, bacteria from the Actinomycetales order are found in soils and aquatic habitats [118,119,120], including domestic water storage containers holding *Ae. aegypti* larvae [121]. Actinomycetes can have antimicrobial properties and larvicidal activity, e.g., the genus *Streptomyces* has recently been indicated as a potential biolarvicide against *Ae. albopictus* [122].

The core microbiota analysis corroborates the influence of the environment on the microbiota of mosquitoes discussed above: the microbiota core of laboratory mosquitoes (F1 + LAB) only had Acetobacteraceae, while the core of field mosquitoes had Bacillaceae, Propionibacteriaceae, Sphingomonadaceae, Streptococcaceae and Vibrionaceae families. These bacterial taxa are commonly found in the environment, associated with the breeding site water or soil (e.g., Bacillaceae, Sphingomonadaceae and Vibrionaceae) [123,124,125]. Some taxa are associated with the human skin and mucosa [126], such as *Propionibacterium* (Propionibacteriaceae) [127] and Streptococcaceae, and could possibly be acquired by *Ae. albopictus* during blood-feeding or contacting materials manipulated by humans.

## 5. Conclusions

This study provides insights into mosquito–microbiota interactions. We explored the variations in *Ae. albopictus* gut bacterial diversity and composition according to collection area and season, origin and population. Our results indicated that mosquitoes collected during the wet season had a more diverse microbiota than those collected during the dry season, and field mosquitoes (irrespective of the collection area) presented a different microbiota composition than those reared in the laboratory (F1 and LAB). Richness and diversity of the gut bacterial communities were greater in field mosquitoes compared with mosquitoes reared for only one generation in the laboratory (F1). We detected significant differences in ZIKV virus infection and dissemination rates between the three different F1 populations of *Ae. albopictus*, which is probably not related to microbiota composition as it was similar between them. A possible co-exclusion interaction between *Wolbachia* and Acetobacteraceae was detected, with the former being absent or undetectable in F1 mosquitoes, while the latter was frequently the most dominant taxa. Future studies concerning mosquito microbe–microbe interactions at the molecular level are necessary to disentangle the network of bacterial interactions that shapes mosquito microbiota. 

## Figures and Tables

**Table 1:** *Aedes albopictus* samples classified according to their collection area, season and generation.

Sample Classification	Collection Area	Collection Season	Origin	Generation
JDG-W	Jardim Guanabara	Wet	Field	F0
JU-W	Jurujuba	Wet	Field	F0
RPC-W	Represa dos Ciganos	Wet	Field	F0
JDG-D	Jardim Guanabara	Dry	Field	F0
JU-D	Jurujuba	Dry	Field	F0
RPC-D	Represa dos Ciganos	Dry	Field	F0
JDG-F1	Jardim Guanabara	LAB *	Laboratory	F1
JU-F1	Jurujuba	LAB *	Laboratory	F1
RPC-F1	Represa dos Ciganos	LAB *	Laboratory	F1
LAB	Laboratory	LAB *	Laboratory	>F30

* Reared under standardized laboratory conditions.

**Table 2:** Variables Used in Diversity Analyses.

Variable	Description	Groups (In Bold)
Area	Samples classified according to collection area (regardless of the collection season) and its respective F1.	JDG (JDG-W + JDG-D), JU (JU-W + JU-D), RPC (RPC-W+RPC-D), JDG-F1, JU-F1, RPC-F1 vs. LAB
Collection season	Period in which insects were collected (regardless of collection area)	WET (JDG-W + JU-W + RPC-W) vs. DRY (JDG-D + JU-D + RPC-D); F1 (JDG-F1 + JU-F1 + RPC-F1) vs. LAB
Origin	Place of mosquito rearing (field or laboratory, regardless collection area and season)	FIELD (JDG-W + JU-W + RPC-W + JDG-D + JU-D + RPC-D), F1 (JDG-F1 + JU-F1 + RPC-F1) vs. LAB
Population	Mosquito population (regardless of collection area, season or rearing place)	JDG (JDG-W + JDG-D + JDG-F1), JU (JU-W + JU-D + JU-F1), RPC (RPC-W + RPC-D + RPC-F1) vs. LAB

**Table 3:** Differentially abundant taxa according to *Aedes albopictus* origin.

Significantly Different Taxa	W *	F1	FIELD	LAB
*Propionibacterium*	202	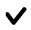	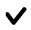	
Acetobacteraceae_unclassified genus	202	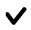		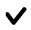
Alphaproteobacteria_unclassified order	200	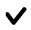		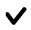
Peptostreptococcaceae_unclassified genus	196		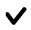	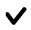
*Rahnella*	188	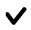		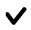
*Cupriavidus*	187			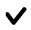
Moraxellaceae_unclassified genus	186			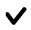
*Pedobacter*	185			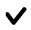
*Reyranella*	182			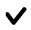
MLE1-12_unclassified family	182			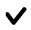
Rhodospirillaceae_unclassified genus	176		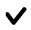	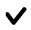
*Hydrocarboniphaga*	175			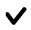
Sphingobacteriales_unclassified family	172		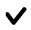	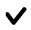

* Considering mosquito origin, a given taxa was considered differentially abundant when W ≥ 172.

**Table 4:** Differentially abundant taxa according to *Aedes albopictus* collection season.

Significantly Different Taxa	W *	WET	DRY	F1	LAB
*Propionibacterium*	202	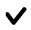	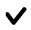	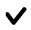	
Acetobacteraceae_unclassified genus	202		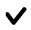	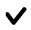	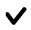
*Wolbachia*	202	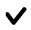	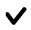		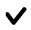
*Methylobacterium*	202	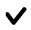			
Peptostreptococcaceae_unclassified genus	201	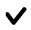	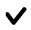		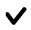
Alphaproteobacteria_unclassified order	199	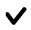		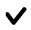	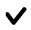
Methylobacteriaceae_unclassified genus	193	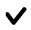	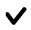		
*Cupriavidus*	190				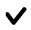
Moraxellaceae_unclassified genus	187				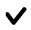
*Pedobacter*	183				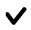
*Reyranella*	183				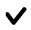
MLE1-12_unclassified family	183				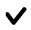
*Rahnella*	177			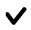	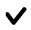
Rhizobiales_unclassified family	177	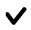	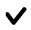		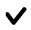
*Hydrocarboniphaga*	177				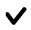
Rhodospirillaceae_unclassified genus	174			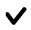	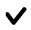
Sphingomonadaceae_unclassified genus	172	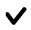	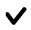	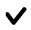	
Sphingobacteriales_unclassified family	171	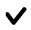			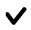
*Bdellovibrio*	171				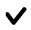
*Ralstonia*	171	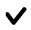		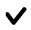	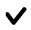
Chitinophagaceae_unclassified genus	169				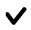

* Considering collection season, a given taxa was considered differentially abundant when W ≥ 169.

**Table 5:** Infection rates (IRs) and dissemination rates (DRs) of ZIKV at 14 and 21 days post-infection (dpi) of *Ae. albopictus* from natural populations collected in locations with different landscapes in Rio de Janeiro.

Population	IR—14dpi (%)	IR—21dpi (%)	DR—14dpi (%)	DR—21dpi (%)
JDG-F1	62.5 (25/40)	40 (4/10)	16 (4/25)	25 (1/4)
JU-F1	30 (12/40)	44.4 (24/54)	16.7 (2/12)	45.8 (11/24)
RPC-F1	85 (34/40)	85.4 (35/41)	64.7 (22/34)	71.4 (25/35)
*Ae. aegypti* (control)	100 (20/20)	95 (19/20)	95 (19/20)	100 (19/19)

The numbers in parentheses indicate ZIKV-positive samples/tested samples.

## Data Availability

Raw read sequences are stored in Sequence Read Archive (SRA) in fastq format (accession: PRJNA951432).

## Supplementary Materials

The following supporting information can be downloaded at: https://www.mdpi.com/article/10.3390/v15061309/s1, Figure S1: Rarefaction curves; Figure S2: Relative abundance of bacterial taxa per sample at phylum level, Figure S3: Relative abundance of bacterial taxa per sample at family level, Table S1: Midgut samples sent for 16S rDNA sequencing; Table S2: Sequencing parameters; Table S3: ASVs removed after decontamination; Table S4: Pairwise comparisons for the four alpha diversity indices; Table S5: Pairwise comparisons for the two beta diversity indices; Table S6: Core microbiota of *Aedes albopictus*.
