# Supplementary figures and images for: Gut Bacterial Diversity of Field and Laboratory-Reared Aedes albopictus Populations of Rio de Janeiro, Brazil
Source: Viruses. 2023 May 31;15(6):1309. doi: 10.3390/v15061309 (PMC10303192; doi:10.3390/v15061309)

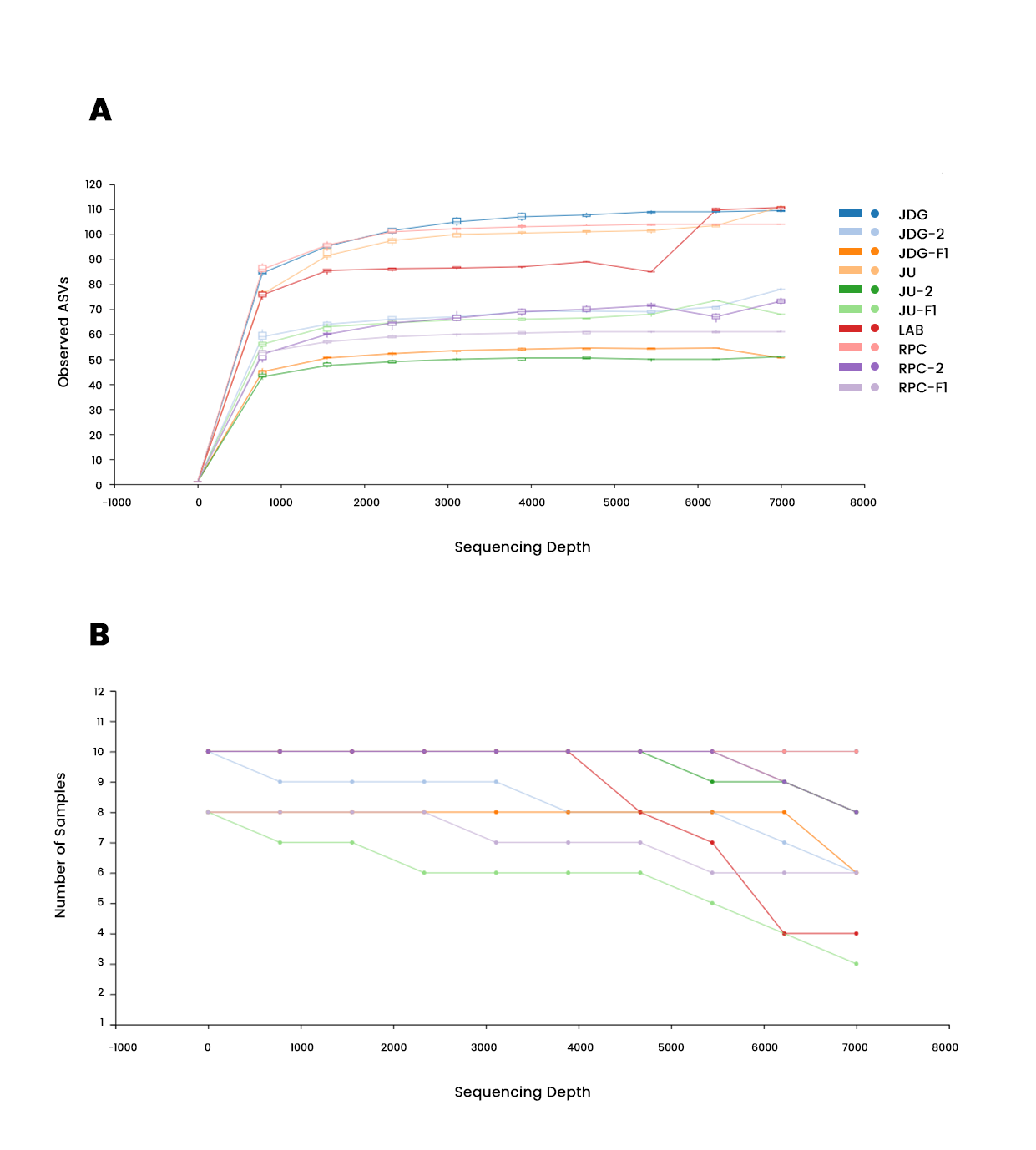

Supplement: Supplementary file 1 [file viruses-15-01309-s001.zip › Figure S1.png]

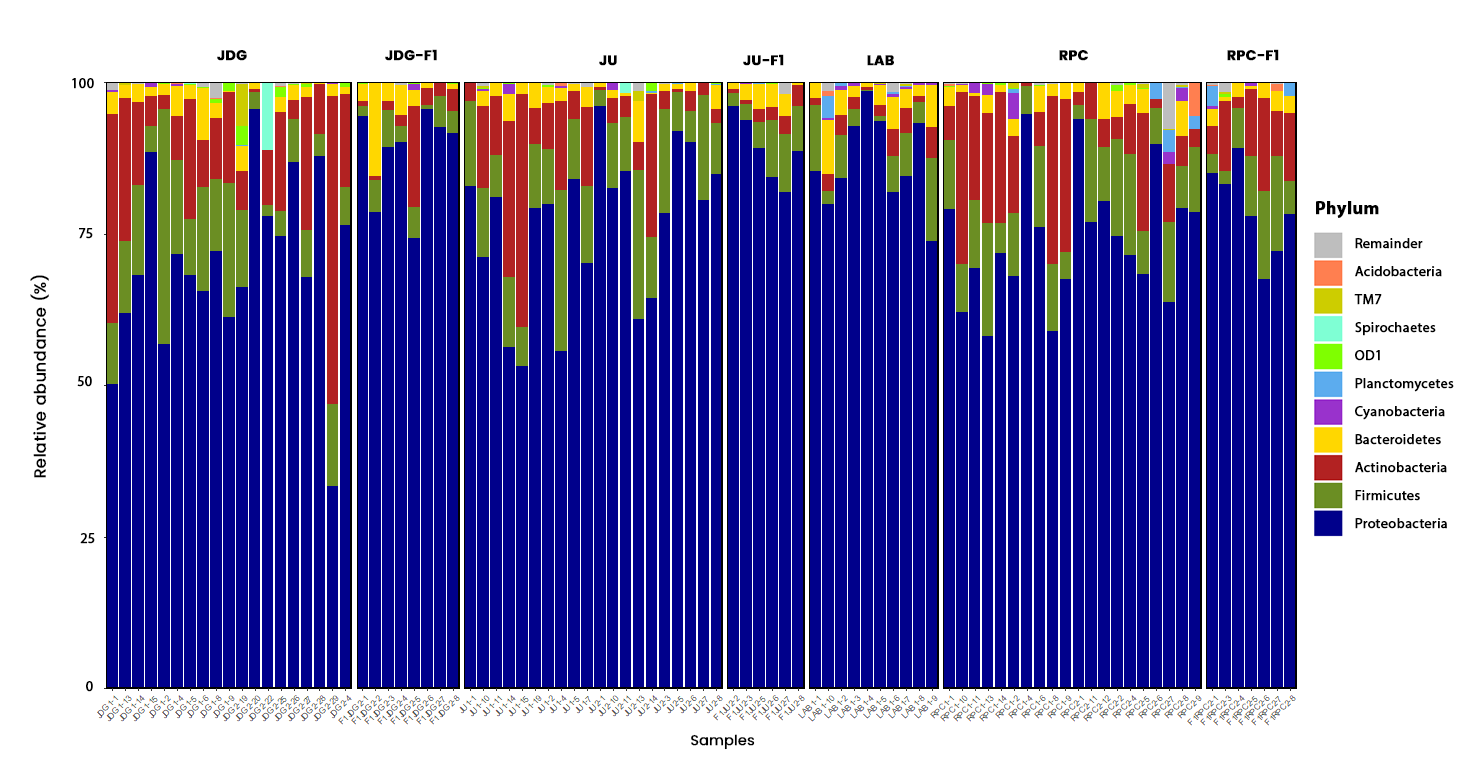

Supplement: Supplementary file 1 [file viruses-15-01309-s001.zip › Figure S2.png]

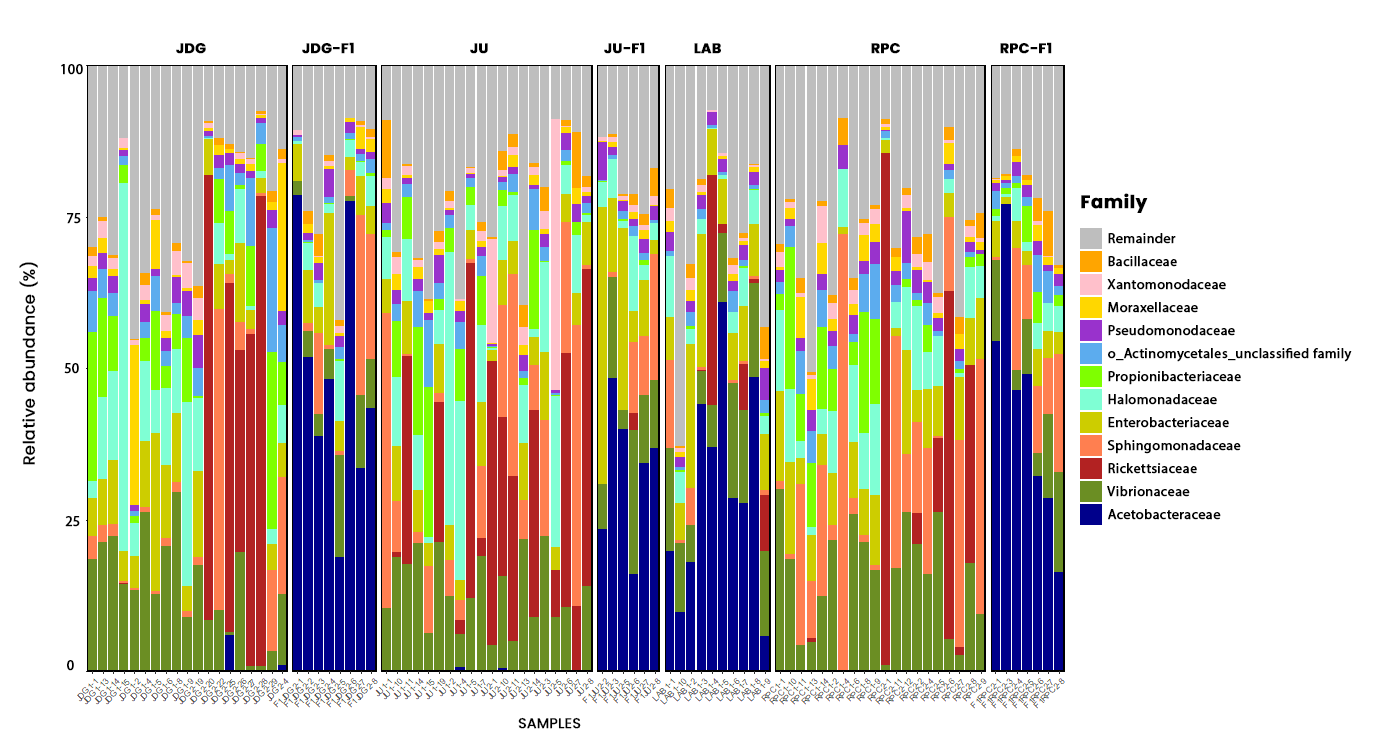

Supplement: Supplementary file 1 [file viruses-15-01309-s001.zip › Figure S3.png]
